# Home-Measured Blood Pressure Is Associated with Handgrip Strength in Patients with Type 2 Diabetes: The KAMOGAWA-HBP Study

**DOI:** 10.3390/jcm10091913

**Published:** 2021-04-28

**Authors:** Tomonori Kimura, Emi Ushigome, Yoshitaka Hashimoto, Naoko Nakanishi, Masahide Hamaguchi, Mai Asano, Masahiro Yamazaki, Michiaki Fukui

**Affiliations:** Department of Endocrinology and Metabolism, Graduate School of Medical Science, Kyoto Prefectural University of Medicine, Kyoto 602-8566, Japan; tomok05@koto.kpu-m.ac.jp (T.K.); y-hashi@koto.kpu-m.ac.jp (Y.H.); naoko-n@koto.kpu-m.ac.jp (N.N.); mhama@koto.kpu-m.ac.jp (M.H.); maias@koto.kpu-m.ac.jp (M.A.); masahiro@koto.kpu-m.ac.jp (M.Y.); michiaki@koto.kpu-m.ac.jp (M.F.)

**Keywords:** handgrip strength, home blood pressure, type 2 diabetes

## Abstract

The association between blood pressure measured at home and handgrip strength in patients with diabetes has not been investigated. Therefore, in this study, we aimed to assess this association among patients with type 2 diabetes. In this cross-sectional study, 157 patients with type 2 diabetes underwent muscle tests and morning and evening blood-pressure measurements at home in triplicate for 14 consecutive days throughout the study period. Univariate and multivariate regression analyses were conducted to analyze the relationship between home blood-pressure parameters and handgrip strength. The average age and hemoglobin A1c of the patients were 70.5 years and 7.1%, respectively. Morning diastolic blood pressure of [β (95% confidence interval; CI): 0.20 (0.03, 0.37)] was associated with handgrip strength in men, while morning systolic blood pressure of [−0.09 (−0.15, −0.04)], morning pulse pressure of [−0.14 (−0.21, −0.08)], and evening pulse pressure of [−0.12 (−0.19, −0.04)] were associated with handgrip strength in women. Home-measured blood pressure was associated with handgrip strength. Sex differences were found in the relationship between home blood-pressure parameters and handgrip strength.

## 1. Introduction

Hypertension is the most common risk factor for cardiovascular diseases (CVDs), and it remains highly prevalent worldwide [1]. Observational data suggest that the treatment of hypertension reduces the risk of diabetic nephropathy and CVD in patients with type 2 diabetes [2]. Home-measured blood pressure (HBP), considered as an important therapeutic parameter, has a strong predictive power for mortality compared with office-measured blood pressure (BP) [3]. Moreover, increased pulse pressure (PP) measured at home is significantly associated with the development of microvascular and macrovascular complications in patients with type 2 diabetes [4]. Patients with type 2 diabetes often suffer from reduced muscle mass and strength [5]. Recent studies have reported that high muscle strength is associated with a lower risk of future CVD events [6]. Moreover, it was reported that increased handgrip strength was associated with lower SBP [7,8,9] and higher DBP [10]. However, no studies have investigated the relationship between HBP parameters and handgrip strength in patients with diabetes. Thus, we aimed to evaluate this association among patients with type 2 diabetes.

## 2. Materials and Methods

### 2.1. Study Design

This study was a cross-sectional study, and baseline data from patients participating in both the type 2 diabetes HBP cohort study (KAMOGAWA-HBP study [3]) and the cohort study on muscle test [11] were used in the present study. Both cohorts included patients who regularly attended the diabetes outpatient clinic at the Kyoto Prefectural University of Medicine Hospital. We evaluated the association of HBP and PP with handgrip strength in patients with type 2 diabetes [11]. All procedures used in this study were approved by the local research ethics committee and were conducted in accordance with the Declaration of Helsinki. Written informed consent was obtained from each patient.

### 2.2. Patients

**Figure 1:**
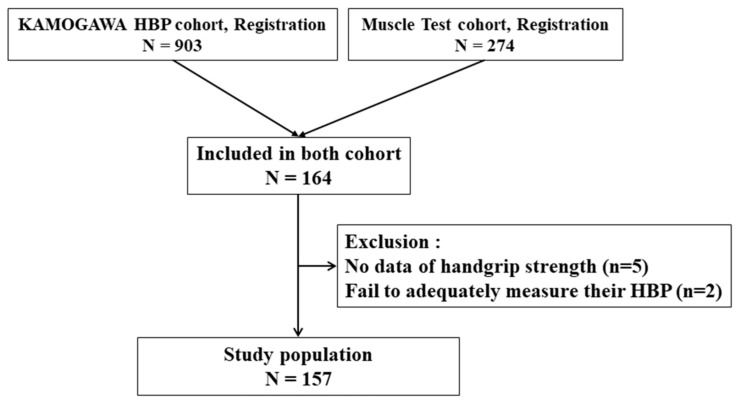
Flow diagram for the KAMOGAWA-HBP cohort.

In this cross-sectional study, 903 and 274 patients with type 2 diabetes underwent blood-pressure measurement at home and a muscle test, respectively, between 2008 and 2017. We included 164 patients who underwent both HBP measurement and handgrip strength measurement. Among them, 2 and 5 patients were excluded because of insufficient data on HBP and unavailability of handgrip strength data, respectively. The study population finally comprised 157 patients (94 men, 63 women, Figure 1). The diagnosis of type 2 diabetes was based on the criteria published by the American Diabetes Association [12].

### 2.3. BP Measurements

The BP of each patient was measured using the HEM-70801C automated BP monitor (Omron Healthcare Co., Ltd., Kyoto, Japan). Morning and evening BP measurements were performed in triplicate for 14 consecutive days. Morning BP was measured within 1 h of waking, before eating breakfast or taking any drugs, while the patient was seated, and had rested for at least 5 min. Evening BP measurement was performed immediately before going to bed. The cuff was placed over the bare upper arm and was positioned at the heart level [13]. We calculated the mean values of the three morning and evening BP measurements. PP was calculated as systolic BP (SBP) minus diastolic BP (DBP).

### 2.4. Measurements of Grip Strength

Handgrip strength was measured twice in the outpatient examination room using a dynamometer (Smedley, Takei Scientific Instruments Co., Ltd., Niigata, Japan) with each hand in a standing position with elbows fully extended. The values were checked by the attending physician, and the maximum value among them was used in this study.

### 2.5. Data Collection

Venous blood was collected after an overnight fast, for biochemical measurements. Hemoglobin A1c (HbA1c) was measured using high-performance liquid chromatography, and results were expressed as a national glycohemoglobin standardization program unit. Body mass index was defined as body weight (kg) divided by the square of the height (m). Patient information, such as age, smoking status (no smoking/current or past smoking), exercise (without exercise habits/with exercise habits), and antihypertensive medication use (without antihypertensive medication/with antihypertensive medication), was collected using a standardized questionnaire at the time of study entry.

### 2.6. Statistical Analysis

Statistical analysis was performed using JMP software version 10.0.2 (SAS Institute Inc., Cary, NC, USA). A *p* value of <0.05 was considered statistically significant. Continuous variables are presented as mean ± standard deviation. Pearson’s correlation analysis was used to investigate the relationship between values of handgrip strength measurement and SBP, DBP, and PP. Univariate and multivariate regression analyses were performed to analyze the relationship between HBP and PP, and handgrip strength. β coefficients were calculated to compare the relationship between HBP and PP, and handgrip strength. To adjust the effects of various factors on handgrip strength, the following known risk factors for handgrip strength were considered as covariates: age, smoking status, exercise, BMI, HbA1c, and antihypertensive medication use [14,15,16,17]. Missing data were excluded from the statistical analyses.

## 3. Results

Clinical characteristics of the study participants are presented in Table 1.

The average (standard deviation) age and HbA1c value were 70.5 (8.5) years and 7.1 (0.8)%, respectively. Eighty-eight patients (56.1%) were treated with antihypertensive drugs. The average values of morning SBP, morning DBP, and morning PP were 133.6 (19.9) mmHg, 75.4 (11.0) mmHg, and 58.2 (15.0) mmHg, respectively. The mean handgrip strength value was 32.4 (7.4) kg in men and 18.8 (4.1) kg in women. In the univariate analysis, morning DBP of [β (95% confidence interval; CI): 0.21 (0.08, 0.34)] and evening DBP of [0.19 (0.04, 0.34)] were associated with handgrip strength in men, while morning SBP of [−0.08 (−0.12, −0.03)], morning PP of [−0.13 (−0.19, −0.08)], evening SBP of [−0.07 (−0.12, −0.02)], and evening PP of [−0.13 (−0.19, −0.07)] were associated with handgrip strength in women (Table 2). In the multivariate analysis, morning DBP of [0.20 (0.03, 0.37)] was associated with handgrip strength in men, while morning SBP of [−0.09 (−0.15, −0.04)], morning PP of [−0.14 (−0.21, −0.08)], and evening PP of [−0.12 (−0.19, −0.04)] were associated with handgrip strength in women (Table 2).

## 4. Discussion

### 4.1. Principal Findings

We assessed, for the first time, the association between HBP parameters and handgrip strength among patients with type 2 diabetes. The main findings of this study were the association of HBP parameters with handgrip strength and the sex differences in the relationship between HBP parameters and handgrip strength. Morning DBP was associated with handgrip strength in men, while morning SBP, morning PP, and evening PP were associated with handgrip strength in women.

### 4.2. Interpretations

In addition to an age-related decrease in activity level and changes in nutrient intake, molecular mechanisms, such as insulin resistance, oxidative stress, chronic inflammation, and changes in sex hormones, have been postulated to contribute to muscle weakness [18]. Type 2 diabetes and hypertension have the same background factors as those mentioned above and are likely to complicate each other. Thus, muscle weakness and BP may be associated in patients with type 2 diabetes. A correlation exists between increased PP and flow-mediated dilatation [19]. The decline in endothelial function is accelerated by aging, hypertension, diabetes mellitus, and dyslipidemia, and it has been shown to affect atherosclerosis development. Higashi, et al. [20] proposed that nitric oxide release may underlie the association between muscle strength and BP. Moreover, age-related loss of nitric oxide synthase in the skeletal muscle was reported to cause reductions in calpain S-nitrosylation, which leads to age-related loss of muscle mass [21].

In this study, the association between HBP and grip strength was stronger in women than in men. This result is consistent with previous reports in which arterial stiffness, measured using cardio-ankle vascular index, was significantly associated with handgrip strength in Japanese non-hypertensive women but not in men [22]. The similarities between the two studies, in which women were older and had higher SBP and lower DBP than men, may have influenced the results. In women, the production levels of estradiol and progesterone declines during menopause [23], and these hormonal changes may also enhance age-related atherosclerosis and muscle weakness [24]. Moreover, a meta-analysis of nearly 10,000 post-menopausal women showed that hormone therapy beneficially affects muscle strength [25]. Estrogen protects skeletal muscle against apoptosis through its effects on heat shock protein and mitochondria [26]. In addition, abnormal inflammatory and satellite cell responses during estrogen deficiency contribute to the loss of muscle strength in women [27].

In the present study, SBP and PP were negatively associated with handgrip strength in women. On the contrary, Taekema et al. [28] found that higher SBP and PP levels were associated with higher handgrip strength in the oldest participants (all 85 years). This is contrary to the results of our study, due to the differences in the patients’ backgrounds. The increase in vascular resistance with aging was speculated to require greater pressure as a mechanism to maintain tissue perfusion, to prevent further damage to ischemic peripheral organs such as skeletal muscles [28].

In this study, DBP was positively associated with handgrip strength in men. Lower levels of DBP were reported to be associated with an increased risk of CVD and death [29]. This may reflect an association between lower DBP levels and more serious comorbidities, including arteriosclerotic vascular disease [30]. Moreover, the absolute values of the β coefficient for morning DBP were higher in men. In the present study, the average of morning DBP was higher in men than in women, which might suggest that men are less likely to have advanced atherosclerosis. Therefore, it is possible that the impact of lower DBP on handgrip strength was stronger in men.

This study has several limitations. First, a cross-sectional design was adopted, which did not show the precise determination of the cause–effect relationship between HBP parameters and handgrip strength. Prospective studies are required to evaluate their association. Second, the small sample size could have influenced the results obtained. Therefore, further studies on bigger groups are needed to fully understand the relationship between HBP and handgrip strength in patients with type 2 diabetes. Third, detailed data on the intake of protein and total energy, which might affect muscle strength, were not available. Fourth, there is no quality control of HBP measurements to ensure that the recommendations given to patients are systematically followed. Fifth, this study included patients with a relatively high capacity for self-care, who were able to self-monitor their HBP. Because of this selection bias, the results of this study may not be applicable to all patients with diabetes. Finally, the mechanism underlying the association between HBP parameters and handgrip strength could not be elucidated because of missing data on inflammatory or oxidative stress markers.

## 5. Conclusions

Our study showed the association between HBP and handgrip strength, particularly in female patients with type 2 diabetes.

## Figures and Tables

**Table 1:** Clinical characteristics of study participants.

	All	Men	Women	*p*
*N*	157	94	63	-
Age (year)	70.5 (8.5)	69.8 (8.9)	71.5 (7.8)	0.201
BMI (kg/m^2^)	23.7 (3.8)	24.0 (3.1)	23.2 (4.4)	0.263
Handgrip strength (kg)	27.0 (9.2)	32.4 (7.4)	18.8 (4.1)	<0.0001
Smoking (never/past or current)	52/105	14/80	38/25	<0.0001
Regular exercise (yes/no)	102/53	47/45	55/8	<0.0001
Antihypertensive drug use (yes/no)	88/69	59/35	29/34	0.539
HbA1c (%)	7.1 (0.8)	7.1 (0.8)	7.1 (0.8)	0.572
Average of morning SBP (mmHg)	133.6 (19.9)	133.4 (18.2)	133.8 (22.4)	0.914
Average of morning DBP (mmHg)	75.4 (11.0)	76.8 (10.9)	73.2 (10.8)	0.043
Average of morning PP (mmHg)	58.2 (15.0)	56.6 (13.6)	60.6 (16.7)	0.107
Average of evening SBP (mmHg)	129.2 (17.9)	128.1 (16.5)	130.8 (19.8)	0.342
Average of evening DBP (mmHg)	70.3 (9.8)	70.7 (10.1)	69.7 (9.4)	0.548
Average of evening PP (mmHg)	58.9 (14.0)	57.4 (12.9)	61.1 (15.4)	0.104

For categorical variables, the number is presented. For continuous variables, the average (standard deviation) is presented. HbA1c, hemoglobin A1c; BMI, body mass index; SBP, systolic blood pressure; DBP, diastolic blood pressure; PP, pulse pressure.

**Table 2:** Unadjusted and adjusted regression analyses on handgrip strength.

	Men		Women	
	Unadjusted	Adjusted ^†^	Unadjusted	Adjusted ^†^
Morning systolic blood pressure	0.04 (−0.05, 0.12)	0.09 (−0.01, 0.19)	−0.08 (−0.12, −0.03)	−0.09 (−0.15, −0.04)
Morning diastolic blood pressure	0.21 (0.08, 0.34)	0.20 (0.03, 0.37)	−0.01 (−0.11, 0.09)	−0.04 (−0.15, 0.08)
Morning pulse pressure	−0.07 (−0.18, 0.04)	0.02 (−0.10, 0.19)	−0.13 (−0.19, −0.08)	−0.14 (−0.21, −0.08)
Evening systolic blood pressure	0.02 (−0.08, 0.11)	0.04 (−0.06, 0.14)	−0.07 (−0.12, −0.02)	−0.06 (−0.12, −0.002)
Evening diastolic blood pressure	0.19 (0.04, 0.34)	0.10 (−0.08, 0.28)	0.04 (−0.07, 0.16)	0.04 (−0.09, 0.17)
Evening pulse pressure	−0.09 (−0.21, 0.03)	0.01 (−0.13, 0.15)	−0.13 (−0.19, −0.07)	−0.12 (−0.19, −0.04)

Data are β coefficients (95% confidence interval). ^†^ Adjusted for age, smoking, exercise, body mass index, hemoglobin A1c, and use of antihypertensive medications.

## Data Availability

All materials are available for use from the corresponding author.
